# Ultrasound- and CT-Guided Medial-to-Lateral Radiofrequency Ablation of the Infraorbital Nerve for Persistent Idiopathic Dentoalveolar Pain: A Trajectory-Based Approach

**DOI:** 10.3390/diagnostics16020254

**Published:** 2026-01-13

**Authors:** Sz-Tsan Wang, Ke-Vin Chang, Wei-Ting Wu, Levent Özçakar

**Affiliations:** 1 Center for Multidisciplinary Pain Management, Dalin Tzu Chi Hospital Buddhist Tzu Chi Medical Foundation, Chiayi 622, Taiwan; curtis210888@gmail.com; 2 Division of Allergy and Immunology and Rheumatology, Dalin Tzu Chi Hospital Buddhist Tzu Chi Medical Foundation, Chiayi 622, Taiwan; 3 Department of Physical Medicine and Rehabilitation, National Taiwan University Hospital, Bei-Hu Branch, No. 87, Nei-Jiang Rd., Wan-Hwa District, Taipei 108, Taiwan; wwtaustin@yahoo.com.tw; 4 Department of Physical Medicine and Rehabilitation, National Taiwan University College of Medicine, Taipei 100, Taiwan; 5 Center for Regional Anesthesia and Pain Medicine, Wang-Fang Hospital, Taipei Medical University, Taipei 116, Taiwan; 6 Department of Physical and Rehabilitation Medicine, Hacettepe University Medical School, Ankara 06100, Turkey; lozcakar@yahoo.com

**Keywords:** ultrasound, ablation radiofrequency, pain, trigeminal neuralgia, odontalgia

## Abstract

Persistent Idiopathic Dentoalveolar Pain (PIDAP) is a persistent idiopathic toothache that frequently remains unresponsive to medical therapy. Precise targeting of the infraorbital nerve is essential for successful intervention, yet anatomical variability often limits the consistency of conventional radiofrequency ablation (RFA). This report describes a medial-to-lateral ultrasound- and computed tomography-guided approach, intended to align with the natural orientation of the infraorbital canal and potentially enhance electrode–nerve contact. A 48-year-old woman with refractory maxillary incisor pain underwent RFA after only transient benefit from a diagnostic nerve block. Ultrasound enabled accurate identification of the infraorbital foramen and confirmed the canal’s medial-to-lateral course, which then guided CT-assisted needle advancement into the orbitomaxillary segment. The patient experienced immediate analgesia. Pain reduction was maintained at the one-month follow-up. At the two-month assessment, although a mild symptom rebound was observed, no procedure-related complications were noted. This trajectory-based medial-to-lateral technique offers an anatomically grounded alternative for infraorbital nerve RFA and may represent a valuable option for refractory PIDAP.

**Figure 1:**
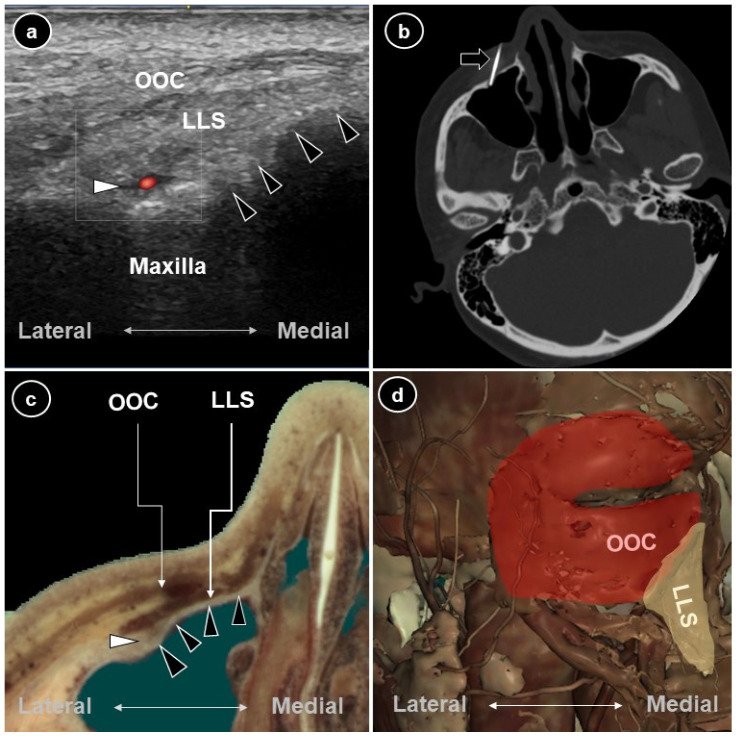
(**a**) Ultrasound image of the left infraorbital foramen showing the medial bony wall (black arrowheads) and the infraorbital foramen (white arrowhead), which transmits the infraorbital artery and nerve. (**b**) CT-guided needle trajectory (black arrow) introduced from medial to lateral to target the orbitomaxillary segment of the infraorbital nerve. (**c**) Cross-sectional cadaveric image from the Visible Human Project^®^ (used with permission from Touch of Life Technologies Inc.) demonstrating the course of the infraorbital canal, consistent with the CT-guided trajectory. (**d**) Corresponding anatomical illustration overlying the infra-orbital foramen including the orbicularis oculi (OOC) and levator labii superioris (LLS). Persistent Idiopathic Dentoalveolar Pain (PIDAP), historically termed atypical odontalgia or phantom tooth pain, is classified by the International Classification of Orofacial Pain as a subtype of idiopathic orofacial pain [1]. It presents as continuous tooth or post-extraction socket pain despite normal clinical and radiographic findings. It occurs in 2.1–6% of patients after endodontic treatment, with a marked female predominance [2]. Its pathophysiology remains unclear, and the diagnosis is made by exclusion in the absence of standardized protocols or definitive treatment guidelines. Current management relies on tricyclic antidepressants, phenytoin, gabapentin, pregabalin, and psychological support [3], however many patients experience inadequate relief or drug intolerance. Radiofrequency ablation (RFA) is an established intervention for trigeminal neuralgia, craniofacial pain, and glossopharyngeal neuralgia. Although peripheral nerve RFA has been effective for infraorbital postherpetic neuralgia [4], it has not been widely adopted for PIDAP. When performing RFA of the infraorbital nerve; ultrasound provides real-time visualization of the superficial cutaneous segment, whereas computed tomography (CT) or fluoroscopic guidance is often required to access the deeper orbitomaxillary portion of the nerve [5]. As needle trajectory affects electrode-nerve contact, careful pre-procedural evaluation is essential. Herein, we describe a medial-to-lateral ultrasound- and CT-guided infraorbital nerve RFA for refractory PIDAP—designed to follow the natural orientation of the infraorbital canal to potentially enhance electrode-nerve contact. A 48-year-old woman without a history of herpes zoster presented with a 6-month history of severe, continuous, throbbing pain at the left frontal incisor—visual analogue scale (VAS) score being 10/10. Crucially, the pain was not precipitated by innocuous stimuli and did not radiate to the surrounding facial skin, differentiating it from classic trigeminal neuralgia. The pain onset was spontaneous, with no identifiable preceding causative event such as trauma, infection, or dental procedures. The condition remained refractory to subsequent extraction of the central incisor and persisted despite multiple trials of tricyclic antidepressants (imipramine, amitriptyline), anticonvulsants (carbamazepine, oxcarbazepine, pregabalin), serotonin–norepinephrine reuptake inhibitors (duloxetine), analgesics (tramadol, oxycodone, etoricoxib, diclofenac), and muscle relaxants (baclofen, tolperisone). A diagnostic infraorbital nerve block had produced marked but transient relief, supporting a peripheral mechanism. Magnetic resonance imaging demonstrated no intracranial lesions and specifically excluded neurovascular compression at the root entry zone of the trigeminal nerve. Botulinum toxin type A injections to the gingival papillae and socket had been ineffective at the one-month follow-up. Notably, the patient was naive to previous neurodestructive interventions or percutaneous procedures targeting the infraorbital foramen. Consequently, infraorbital nerve RFA was planned. Real-time ultrasound guidance was established using an L8-18i hockey-stick transducer (Logiq S8 ultrasound system, GE HealthCare Technologies Inc, Chicago, IL, USA). On ultrasound, the infraorbital foramen was visualized deep to the levator labii superioris and orbicularis oculi muscles. Its concave bony floor was seen to merge medially with the maxillary cortex, while a canal extended laterally (Figure 1a). In accordance with this anatomical configuration, a medial-to-lateral in-plane approach was adopted (Figure 1b). CT confirmed accurate needle placement within the infraorbital foramen, with the electrode tip contacting the superior wall of the maxillary sinus. Sensory stimulation reproduced concordant paresthesia. Conventional RFA at 80 °C for 120 s was performed. The patient experienced immediate analgesic benefit, with the VAS score decreasing from 10 to 2. At the one-month follow-up, pain reduction was maintained. At the two-month assessment, although a mild symptom rebound was observed, no procedure-related complications—such as ophthalmological injury, corneal damage, or facial motor denervation—were noted. The patient reported anticipated paresthesia affecting the central incisor, lateral incisor, and canine (V2 distribution), confirming accurate targeting of the infraorbital nerve. A critical step in our diagnostic workup was the use of a prognostic local anesthetic (LA) nerve block. The role of LA blocks in PIDAP is complex, as the condition involves both peripheral and central mechanisms. While some studies, such as those by Graff-Radford and Solberg [6], found no significant benefit of LA in these patients—suggesting a predominant central sensitization mechanism—others have reported contrasting results. Vickers et al. [7] and List et al. [8] demonstrated that LA blocks could provide significant, albeit transient, pain relief in patients with PIDAP. Taken together, these heterogeneous findings suggest that LA blocks may transiently alleviate pain in patients with peripheral or neuropathic contributions, whereas limited or absent responses may reflect predominant central sensitization. Further studies are needed to clarify the clinical implications of LA responses in PIDAP. While cross-innervation of the maxillary central incisors is described in the literature, the substantial relief provided by the unilateral diagnostic block in this case confirmed the ipsilateral infraorbital nerve as the primary mediator of nociception. RFA is supported by evidence for atypical facial pain and infraorbital neuralgia [9,10]. Lesion proximity is a critical determinant of both efficacy and safety. In cases of trigeminal neuralgia involving the maxillary division (V2), lesioning at the foramen rotundum or within the pterygopalatine fossa has demonstrated superior long-term outcomes and fewer complications compared with more proximal gasserian ganglion ablation performed through the foramen ovale [9,11]. Similarly, our aim was to target the infraorbital nerve trunk near its exit point to optimize contact and lesion quality. The infraorbital canal typically follows an anterior–medial to posterior–lateral trajectory, consistent with the slope of the maxilla and maxillary sinus (Figure 1c). High-resolution ultrasound clearly delineated the overlying musculature (Figure 1d) [12], facilitating accurate localization of the infraorbital foramen. It also confirmed the canal’s spatial orientation, supporting the medial-to-lateral trajectory subsequently used under CT guidance. The rationale for this trajectory is grounded in the biophysics of radiofrequency ablation. The efficacy of RFA is fundamentally dependent on the proximity of the active electrode tip to the target nerve, as thermal lesion size and the probability of successful neurolysis decrease rapidly with distance from the probe [13,14]. Guidelines from the American Society of Pain and Neuroscience emphasize that anatomical variations can lead to suboptimal placement; therefore, precise verification is essential [15]. By utilizing an ultrasound-first approach followed by CT confirmation, we achieved precise spatial proximity to the infraorbital foramen. Unlike perpendicular or lateral-to-medial approaches that may result in limited point-contact, the medial-to-lateral trajectory aligns the needle coaxially with the infraorbital canal. This alignment theoretically maximizes the infraorbital nerve, ultrasound-nerve interface, ensuring that a longer segment of the nerve trunk lies within the effective thermal zone. Although the clinical reproducibility of this strategy requires further validation in larger cohorts, this image-guided precision represents a significant technical advantage for optimizing lesion quality. Anatomical variation is an important consideration. The infraorbital canal and foramen vary in shape, orientation, number, and spatial relation to bony landmarks. Bifid or atypically oriented canals may increase procedural risk if trajectory planning relies solely on classical textbook knowledge [11,16]. Sensory innervation to the frontal incisors arises from the maxillary division (V2) of the trigeminal nerve. It passes through the foramen rotundum, pterygopalatine fossa, and infraorbital canal before emerging at the infraorbital foramen—typically 6–8 mm below the infraorbital rim and 23–27 mm lateral to the midline [16,17]. Pre-procedural ultrasound examination is therefore crucial to define the morphology and direction of the infraorbital foramen prior to intervention. In conclusion, this case describes a trajectory-based medial-to-lateral ultrasound- and CT-guided approach for infraorbital nerve RFA in refractory PIDAP. By aligning with the anatomical course of the infraorbital canal, this technique aims to enhance electrode–nerve contact and procedural safety. This approach represents a feasible technical alternative and may also represent a valuable option for patients with persistent maxillary-division pain. However, as this report is based on a single case, comparative efficacy against standard approaches cannot be established without further investigation. Finally, this report is limited by the absence of formal longitudinal follow-up data. Although psychological counseling was provided, quantitative psychometric assessments were not systematically recorded. Future studies should incorporate standardized dual-axis assessments to more comprehensively evaluate psychosocial factors in persistent idiopathic dentoalveolar pain.

## Data Availability

The raw data supporting the conclusions of this article will be made available by the authors on request.
